# Driving-related behaviors, attitudes, and perceptions among Australian medical cannabis users: results from the CAMS 20 survey

**DOI:** 10.1186/s42238-023-00202-y

**Published:** 2023-09-06

**Authors:** Thomas R. Arkell, Sarah V. Abelev, Llewellyn Mills, Anastasia Suraev, Jonathon C. Arnold, Nicholas Lintzeris, Iain S. McGregor

**Affiliations:** 1 Centre for Mental Health and Brain Science, Swinburne University of Technology, Melbourne, VIC Australia; 2 Lambert Initiative for Cannabinoid Therapeutics, Brain and Mind Centre, University of Sydney, Sydney, NSW Australia; 3 Drug and Alcohol Services, South East Sydney Local Health District, Sydney, NSW Australia; 4 Department of Addiction Medicine, Faculty Medicine and Health, University of Sydney, Sydney, NSW Australia; 5 Faculty of Science, School of Psychology, University of Sydney, Sydney, NSW Australia; 6 Discipline of Pharmacology, Sydney Pharmacy School, Faculty of Medicine and Health, University of Sydney, Sydney, NSW Australia

**Keywords:** Medical cannabis, THC, CBD, Cannabinoid, Driving, Road safety

## Abstract

Road safety is an important concern amidst expanding worldwide access to legal cannabis. The present study reports on the driving-related subsection of the Cannabis as Medicine Survey 2020 (CAMS-20) which surveyed driving-related behaviors, attitudes, and perceptions among Australian medical cannabis (MC) users. Of the 1063 respondents who reported driving a motor vehicle in the past 12 months, 28% (297/1063) reported driving under the influence of cannabis (DUIC). Overall, 49–56% of respondents said they typically drive within 6 h of MC use, depending on the route of administration (oral or inhaled). Non-medical cannabis (NMC) was perceived to be more impairing for driving than MC. Binary logistic regression revealed associations between likelihood of DUIC and (1) inhaled routes of cannabis administration, (2) THC-dominant products, (3) illicit rather than prescribed use, (4) believing NMC does not impair driving, and (5) not being deterred by roadside drug testing. Overall, these findings suggest there is a relatively low perception of driving-related risk among MC users. Targeted education programs may be needed to highlight the potential risks associated with DUIC, and further research is needed to determine whether driving performance is differentially affected by MC and NMC.

**Supplementary Information:**

The online version contains supplementary material available at 10.1186/s42238-023-00202-y.

## Introduction

Prescriptions for medical cannabis (MC) have increased tremendously in Australia since legalization in November 2016. This upward trajectory has been driven by streamlining of patient access pathways and increasing numbers of medical practitioners prescribing MC products (MacPhail et al. 2022). Despite improved access to MC, it remains an offence in Australia to drive with the presence of Δ^9^-tetrahydrocannabinol (THC) in one’s blood or oral fluid. Under the “roadside drug testing” (RDT) program, police can request an oral fluid sample from a driver at any time and have this sample screened for THC at the roadside. With the exception of one jurisdiction (Tasmania), a valid prescription for MC does not provide an exemption against prosecution in the case of a driver testing positive for THC. These restrictions on driving are a considerable barrier to patient access and a complex road safety issue (Ramaekers 2018).

Research to date demonstrates that THC can impair driving for 3–8 h in occasional users, with duration of impairment dependent on dose, route of administration and individual tolerance. While higher THC doses increase the risk of impairment (Ramaekers et al. 2000; Cook et al. 2020), experimental studies indicate that the overall driving impairment caused by cannabis is modest, and similar in magnitude to that seen in drivers with a blood alcohol concentration (BAC) of around 0.05% (Arkell et al. 2020a; Ramaekers et al. 2000). However, a notable caveat is that most studies of impairment have utilized young, healthy, occasional cannabis users with limited driving experience and THC doses intended to produce intoxication and impairment. Whether patients who are prescribed MC to manage a health condition exhibit similar driving impairment has yet to be established. While several US studies have looked at the link between the relaxation of cannabis laws and road traffic injuries, findings are mixed and inconclusive (Cook et al. 2020; Fink et al. 2020; Sevigny 2018; Santaella-Tenorio et al. 2017).

The Cannabis as Medicine Survey (CAMS) has explored the landscape of MC use in Australia every 2 years since 2016. In the 2018 iteration of the survey (CAMS-18), 34.6% of respondents said they typically drive within 3 h of cannabis use, which is when impairment is most likely to occur (Arkell et al. 2020b). Most respondents thought that MC did not affect their driving ability, and most denied adverse effects of their cannabis use on speeding, risk taking, reaction time, attentiveness or lane departures. In addition, heavier cannabis use and greater confidence in self-assessment of driving ability were associated with increased likelihood of driving shortly after cannabis use. Since then, a recent Canadian analysis reported that MC users were almost twice as likely to engage in driving under the influence of cannabis (DUIC) as NMC users, even after controlling for confounders such as frequency of use (Wickens et al. 2022). Also notable was a recent US survey where the likelihood of driving while impaired among frequent cannabis users (≥ 20 days per month) was similar in states with legal MC and states without legal cannabis (Dutra, et al. 2022).

One limitation with our previous CAMS-18 survey was that only 2.4% of respondents (*n* = 25) reported accessing legally prescribed MC. Most were self-medicating with illicit products obtained from a “dealer” or through family or friends. In this more recent iteration of CAMS (CAMS-20), 62.4% of respondents (*n* = 999) were still sourcing their MC via illicit routes while 37.5% of respondents (*n* = 601) were accessing prescribed MC in line with the striking increase in prescribing since 2018 (MacPhail et al. 2022). The substantial increase in the proportion of patients using prescribed MC in CAMS-20 offers an opportunity to reassess the prevalence of self-reported DUIC behaviors in the CAMS-20 population and investigate whether attitudes and beliefs toward DUIC have changed in more recent years. The term “medical cannabis” (MC) used in this paper refers to any prescribed (legal) or illicit cannabis product (including plant matter) used to treat or alleviate the symptoms of a self-identified health condition.

## Methods

The Cannabis as Medicine Survey 2020 (CAMS-20) was an anonymous, cross-sectional online survey examining MC use in Australian adults. Survey data were collected between September 2020 and January 2021. Eligible respondents to the survey were ≥ 18 years of age and had used prescribed or illicit cannabis to treat a medical condition within the past 12 months. The survey included questions on demographics, patterns of MC use including formulations and administration methods, perceived benefits and side effects of MC use, attitudes towards MC-related contemporary issues and general wellbeing and driving. Full details of the questionnaire are available elsewhere (Lintzeris et al. 2022).

### Driving outcome measures

A series of questions relating to driving behaviors and attitudes towards DUIC were included as the penultimate section of the CAMS-20 survey. The specific questions asked in the driving section are presented in Additional file 1: Appendix A. In brief, if a respondent indicated they had driven a motor vehicle in the past 12 months, they were presented with questions relating to their history of DUIC, typical wait times between using cannabis and driving, and whether they had encountered roadside drug testing. DUIC was defined as self-reported driving while under the influence of cannabis (i.e., while “high”), regardless of whether it was medical or not. Respondents were then asked to respond to statements about the specific impact of MC on their reaction time, focus, lane keeping, speed limit adherence, and risky driving behaviors. Responses were captured on 5-point Likert scales ranging from “strongly disagree” to “strongly agree.” Respondents were also asked whether they believe (1) MC and (2) non-medical cannabis (NMC) impair their driving ability.

### Statistical analysis

Respondents who had driven a motor vehicle in the past 12 months were included in the analyses. Data were analysed using SPSS version 26 (IBM Corp., Armonk, NY). Respondents were split into three groups according to the legal status of the cannabis they used: those who used prescribed MC only (prescribed only), those who used illicit MC only (illicit only), and those who used both prescribed and illicit MC (dual prescribed and illicit). Binary logistic regression was used to investigate the relationship between past-year DUIC (yes/no) and the following variables: age, gender, education, employment status, prescribed/illicit/dual use, route of administration, cannabinoid profile, belief in whether MC/NMC impairs driving, and deterrent effect of RDT. These variables were selected based on their relevance to DUIC and with the intention of comparing CAMS-20 results against findings from CAMS-18 (Arkell et al. 2020b). The threshold for statistical significance was *p* < 0.05.

## Results

Table 1 summarizes the demographics, characteristics, and MC use patterns of the 1063 respondents who reported driving a motor vehicle in the past 12 months. Respondents (*n* = 460, 43.3% female) had a mean (SD) age of 46.3 (13.5). Most had completed a university degree (*n* = 439, 41.3%) or a trade/vocational certificate (*n* = 429, 40.4%), and most were engaged in either full or part time work (*n* = 558, 43.6%). Most were using illicitly sourced cannabis only (*n* = 687, 64.6%),124 (11.7%) were using prescribed cannabis only, and 252 (23.7%) had used both illicit and prescribed cannabis in the past 12 months (“dual” users). The most common conditions for use included pain (*n* = 181, 32.7%), sleep (*n* = 154, 27.8%), and mental health (*n* = 136, 24.6%). Inhaled routes of administration were most common among illicit only users (61.6% [*n* = 416] vs. 11.5% of prescribed only users [*n* = 14] and 37.9% of dual users [*n* = 94]), while conversely, orally administered products were most common among prescribed only users (88.5% [*n* = 108] vs 38.4% of illicit only users [*n* = 259] and 24.6% of dual users [*n* = 61]).

**Table 1:** Respondent demographics, characteristics, and medicinal cannabis use patterns

	**Illicit only (*n* = 687)**		**Prescribed only (*n* = 124)**		**Dual (*n* = 252)**		**Total (*n* = 1063)**	
	***n***	**%**	***n***	**%**	***n***	**%**	***n***	**%**
**Age (mean, SD)**	47.2 (13.9)		48.6 (13.2)		42.8 (11.7)		46.3 (13.5)	
**Gender**								
Female	302	44.0	72	58.1	86	34.1	460	43.3
Male	379	55.2	52	41.9	163	64.7	594	55.9
Other	6	0.01	0	0.0	3	1.2	9	0.01
**Route of administration **								
Inhaled	416	61.6	14	11.5	94	37.9	524	50.1
Oral	259	38.4	108	88.5	61	24.6	428	41.0
Oral + inhaled	0	0	0	0	93	37.5	93	8.9
**Cannabinoid**								
THC dominant	200	29.4	19	15.3	120	57.2	339	33.5
Balanced	122	18.0	47	37.9	0	0	169	16.7
CBD dominant	147	21.6	57	45.9	18	8.6	222	21.9
Unknown	210	30.9	1	0.8	72	34.3	283	27.9
**Education**								
Primary	5	0.7	1	0.9	2	0.8	8	0.8
High school	133	19.5	20	18.3	34	13.7	187	17.6
Trade	259	38.0	38	34.9	107	43.1	429	40.4
Undergrad	193	28.3	28	25.7	68	27.4	150	14.1
Postgrad	91	13.4	22	20.2	37	14.9	289	27.2
**Employment**								
FT/PT	361	52.5	57	46.0	140	55.6	558	43.6
Unemployed	59	8.6	12	9.7	28	11.1	99	7.7
Home/student	68	9.9	12	9.7	16	6.4	96	7.5
Retired	85	12.4	19	15.3	18	7.1	122	9.5
Disability	114	16.6	24	19.4	50	19.8	406	31.7
**General condition**								
Cancer	70	4.4	6	2.4	14	2.6	10	1.8
Gastrointestinal	133	8.3	17	6.9	30	5.6	29	5.2
Mental health	347	21.7	39	15.9	121	22.7	136	24.6
Neurological	103	6.4	20	8.1	30	5.6	43	7.8
Pain	508	31.7	95	38.6	178	33.3	181	32.7
Sleep	440	27.5	69	28.0	161	30.1	154	27.8

% as a proportion of all responses. Respondents could select multiple general conditions

### Driving behaviors and attitudes toward DUIC

**Fig. 1:**
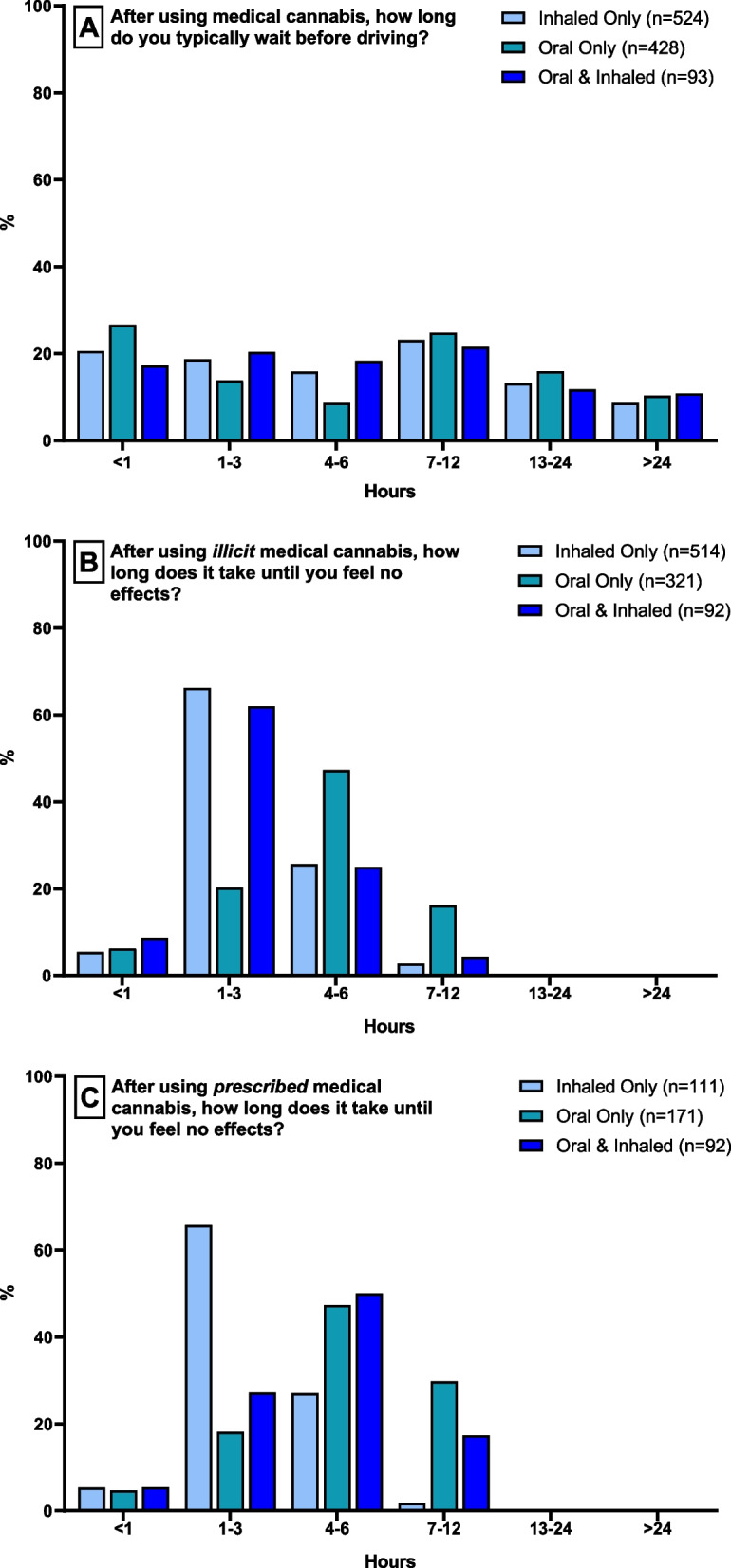
Panel **A** shows the length of time that respondents typically wait before driving after using medical cannabis. Panels **B** and **C** shows respondents’ experience of the duration of effects following illicit medical cannabis use (panel **B**) and prescribed medical cannabis use (panel **C**). All responses are stratified by route of administration

Figure 1 shows respondents’ self-reported wait time between consuming MC and driving (panel A) and self-reported duration of MC effects (panels B–C). Irrespective of whether MC was illicit (panel B) or prescribed (panel C), most respondents using inhaled products only or both inhaled and oral products estimated the duration of effects to be within 1–3 h (*n* = 340, 66.15% of respondents using illicit inhaled products, *n* = 57, 61.96% of respondents using both inhaled and oral products, *n* = 73, 65.77% of respondents using prescribed inhaled products, and *n* = 25, 27.17% of respondents using prescribed inhaled and oral products). Most respondents using oral products estimated the duration of cannabis effects to be within 4–6 h (*n* = 152, 47.35% of respondents using illicit oral products; *n* = 81, 47.37% of respondents using prescribed oral products). For those using inhaled products only, *n* = 206 (20.6%) said they typically drive within 3 h of use, and *n* = 289 (55%) said they typically drive within 6 h of use. Less than 10% (*n* = 45) reported waiting more than 24 h before driving. A similar pattern was observed for those using oral products only (driving within 3 h of use: *n* = 173, 40.4%; driving within 6 h of use: *n* = 210, 49%), and both inhaled and oral products (driving within 3 h of use: *n* = 35, 37.6%; driving within 6 h of use: *n* = 52, 55.9%).

Table 2 summarizes attitudes towards DUIC as a prelude to the regression analysis. DUIC during the previous 12 months was reported by 27.8% (*n* = 295) of respondents who said they did so very rarely (*n* = 82; 27.8%), rarely (*n* = 45; 15.3%), sometimes (*n* = 64; 21.7%), often (*n* = 50; 16.9%), or very often (*n* = 54; 18.3%). A small majority (*n* = 590; 55.5%) indicated that RDT deters them from driving after using MC. Over half (50.5%; *n* = 536) thought that NMC impairs driving ability while only 22.1% (*n* = 234) thought that MC impairs driving ability.

**Table 2:** Respondent attitudes and experiences around driving under the influence of cannabis (DUIC)

		Illicit only		Prescribed only		Dual		Total	
		***n***	**%**	***n***	**%**	***n***	**%**	***n***	**%**
In the past 12 months, did you ever drive while under the influence of cannabis?	No	481	70.0	113	91.1	174	69.0	768	72.2
	Yes	206	30.0	11	8.9	78	31.0	295	27.8
Does the presence of roadside drug testing deter you from driving after you have consumed medicinal cannabis?	No	304	44.3	60	48.4	109	43.3	473	44.5
	Yes	383	55.7	64	51.6	143	56.7	590	55.5
Do you think non-medical ('recreational') cannabis impairs your driving ability?	No	361	52.5	37	29.8	128	51.0	526	49.5
	Yes	326	47.5	87	70.2	123	49.0	536	50.5
Do you think medicinal cannabis impairs your driving ability?	No	525	76.4	105	84.7	198	78.6	828	77.9
	Yes	162	23.6	19	15.3	54	21.4	235	22.1

### Perceived effects of MC on driving

**Fig. 2:**
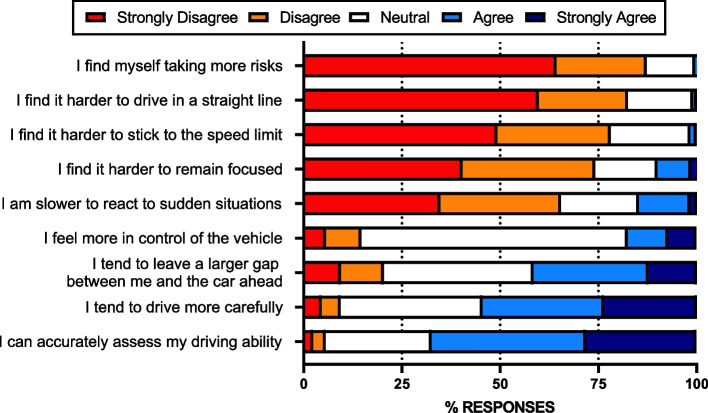
Respondents’ endorsement of various statements describing driving-related behaviors following their use of medical cannabis

Figure 2 shows the extent to which respondents agreed or disagreed with statements relating to cannabis effects on driving after considering how their MC use ordinarily affects them. Only a small minority of respondents said that they take more risks (strongly agree or agree: *n* = 5, 0.5%), find it harder to drive in a straight line (strongly agree or agree: *n* = 10, 0.9%), or have trouble adhering to the speed limit (strongly agree or agree: *n* = 17, 1.6%). Relatively few respondents said they find it harder to remain focused (strongly agree or agree: *n* = 107, 10.1%) or are slower to react to sudden situations (strongly agree or agree: *n* = 156, 14.7%). Most respondents agreed that they tend to drive more carefully (strongly agree or agree: *n* = 579, 54.5%), can accurately assess their driving ability (strongly agree or agree *n* = 717, 67.4%), and tend to leave a larger gap between them and the car ahead (strongly agree or agree: *n* = 441, 41.5%). Respondents were mostly neutral as to whether they felt more in control of the vehicle (*n* = 720, 67.7%).

### Binary logistic regression

As shown in Table 3, six variables were associated with DUIC. Respondents who reported DUIC were more likely to use illicit (rather than prescribed) products (OR = 2.18, 95% CI 1.06–4.51, *p* = 0.035), were less likely to use oral products (OR = 0.61, 95% CI 0.39–0.96, *p* = 0.031) or CBD dominant products (OR = 0.37, 95% CI 0.19–0.70, *p* = 0.002), used MC more frequently throughout the day (OR = 1.09, 95% CI 1.05–1.13, *p* < 0.001), believed that recreational cannabis does not impair driving ability (OR = 3.53, 95% CI 2.26–5.51, *p* < 0.001), and were not deterred by RDT (OR = 1.97, 95% CI 1.44–2.69, *p* < 0.001).

**Table 3:** Binary logistic regression model assessing likelihood of driving under the influence of cannabis (DUIC)

Variable	Category	*Ref*	OR	Lower 95% CI	Upper 95% CI	*p*
Age			0.44	1.00	0.98	0.442
Gender	Male	*Female*	1.11	0.80	1.54	0.529
Education	Primary	*Undergrad*	2.74	0.55	13.74	0.221
	Secondary	*Undergrad*	1.13	0.71	1.80	0.613
	Trade	*Undergrad*	**1.61**	**1.10**	**2.35**	**0.015**
	Postgrad	*Undergrad*	0.85	0.50	1.44	0.541
Employment	Not employed	*FT/PT*	0.98	0.58	1.66	0.848
	Other	*FT/PT*	0.97	0.68	1.38	0.940
User type	Illicit only	*Prescr. only*	**2.06**	**1.01**	**4.22**	**0.049**
	Dual	*Prescr. only*	1.73	0.78	3.86	0.178
Admin	Oral only	*Inhaled only*	**0.51**	**0.33**	**0.78**	**0.002**
	Oral, inhaled	*Inhaled only*	0.92	0.50	1.70	0.799
Cannabinoid	Balanced	*THC dom*	0.62	0.38	1.03	0.063
	Variable	*THC dom*	0.77	0.54	1.10	0.146
	CBD dom	*THC dom*	**0.33**	**0.18**	**0.63**	** < 0.001**
NMC impairs	No	*Yes*	**3.83**	**2.47**	**5.95**	** < 0.001**
MC impairs	No	*Yes*	0.91	0.54	1.55	0.733
RDT deter	No	*Yes*	**1.90**	**1.40**	**2.58**	** < 0.001**

*Abbreviations:* MC impairs, “Do you believe medicinal cannabis impairs driving?”; NMC impairs, “Do you believe recreational cannabis impairs driving?”; RDT deter, “Does the presence of RDT (roadside drug testing) deter you from DUIC?”; bolded ORs are significant at the *p* < 0.05 level

## Discussion

This study was designed to assess driving-related behaviors and attitudes among a convenience sample of Australian MC users recruited as part of our larger CAMS-20 survey. One of the biggest differences between the present iteration of this survey and prior CAMS surveys (CAMS-16 (Lintzeris et al. 2018) and CAMS-18 (Lintzeris et al. 2020)) is the number of respondents using prescribed MC products. While this number was very small in CAMS-18 (*n* = 25) and even smaller in CAMS-16 (*n* = 1), it was much greater here (*n* = 376, including both dual and prescribed only categories), signifying an important transition underway in the Australian community from illicit towards legal prescribed MC products (MacPhail et al. 2022).

While there were relatively few differences in demographic characteristics across the different surveys, there were several notable differences in cannabis use characteristics. Perceived knowledge around the cannabinoid profile of the product being used was far greater in the present survey, even among illicit only users, with fewer participants reporting uncertainty around cannabinoid profile (30.9%) when compared with the total of 48% in CAMS-18 (Lintzeris et al. 2020). This presumably reflects the certainty of cannabinoid content that comes with legally prescribed products. The use of orally administered products was also higher here than previously, even among illicit only users (38.4 vs 26.6%). Among those using only prescribed products, the majority (88.5%) reported using orally administered products, with only a small number (11.5%) using inhaled products. These figures are consistent with general prescribing trends in Australia as reported by the Therapeutic Goods Administration (Therapeutic Goods Adminsitration 2022), although it is worth noting that prescriptions for inhaled products are steadily increasing (MacPhail et al. 2022).

A key safety concern with MC products is that driving shortly after the use of THC-dominant products may impair driving and therefore increase crash risk. In a recent Canadian survey, 23% of people who had used cannabis in the past 12 months reported driving within 2 h of smoking or vaporizing cannabis at some point in their life, while 14% reported driving within 4 h of ingesting cannabis at some point in their life (Government of Canada 2022). In our analysis, around half of the current cohort endorsed the idea that NMC use can impair driving and a large majority reported that the product they used had effects that lasted for 1–3 or 4–6 h. This interval of up to 6 h is the generally acknowledged window in which driving and cognitive impairment is most likely to be observed in occasional cannabis users. Notably, around 50% of all respondents in the survey typically drove within this 6-h window after use. Despite this, respondents generally thought that their MC use did not impair their ability to drive and less than 30% of illicit users and dual users admitted to driving while “high.”

Very few agreed that their MC caused them to take more risks, made it harder to drive in a straight line, or adhere to the speed limit. Approximately 10% of respondents did, however, indicate that they find it harder to remain focused while driving after using MC, and 14.7% reported being slower to react to sudden situations. Almost 50% of respondents said that they tend to leave a larger gap between their car and the car ahead and that they tend to drive more carefully with cannabis. This pattern of behavior is often observed in experimental studies and is thought to reflect an attempt to compensate for perceived impairment (Hartman and Huestis 2013). As with CAMS-18, most respondents felt they could accurately assess their driving ability after using MC. While the validity of this claim has yet to be empirically tested in patient populations, there is evidence to suggest that individuals are generally aware of their impairment after consuming cannabis (Hartman and Huestis 2013). At the same time, a recent study in healthy volunteers who were regular cannabis users showed a reduction in perceived driving impairment at 1.5 h relative to 0.5 h after smoking cannabis containing either 5.9 or 13.4% THC, even though there was no objective improvement in driving performance over this 1-h period (Marcotte et al. 2022).

Binary logistic regression analysis revealed several important predictors of DUIC, including legality of use, route of administration, cannabinoid profile, belief in whether NMC (but not MC) cannabis impairs driving, the deterrent effect of RDT, and frequency of cannabis use per day. Respondents who were using illicit products only were twice as likely to engage in DUIC relative to those using prescribed products only, and belief that NMC does not impair driving was associated with an almost 4-fold increase in the likelihood of DUIC. The use of orally administered products was associated with a close to 40% reduction in the likelihood of DUIC relative to inhaled only products, while use of CBD-dominant products was associated with a close to 60% reduction in DUIC relative to use of THC-dominant products. This latter finding is perhaps unsurprising given that CBD is non-intoxicating and does not impair driving performance (McCartney, et al. 2022; Arkell et al. 2020c) and noting that the question around DUIC specifically asked respondents if they had driven while “under the influence (i.e., while high)” in the past 12 months. Unlike CAMS-18, where unemployment was associated with a 4.7-fold increase in the likelihood of DUIC, employment status was not a significant predictor of DUIC in CAMS-20. This previous finding may therefore have been an artefact of the small number of respondents who were unemployed (*n* = 26), as we hypothesized at the time (Arkell et al. 2020b).

Consistent with the CAMS-18 survey (Arkell et al. 2020b), we observed a significant deterrent effect of RDT with most respondents saying that RDT deterred them from driving after consuming MC. This deterrent effect was similar across users of illicit only products (81.8%), prescribed only products (81%), and dual users (82.1%). Respondents who were not deterred by the presence of RDT were almost twice as likely to engage in DUIC. The legality of the product used may therefore have little influence on willingness to drive with THC in one’s system which likely reflects the fact that current laws do not discriminate between users of prescribed and illicit products. It is worth noting that the population sampled here may have excluded patients who are deterred from using MC altogether by current driving laws.

One key question with tangible implications for policy is whether patients who use MC as prescribed are less impaired than individuals who use cannabis for other reasons. Recent reviews provide little evidence of impairment in those using stable doses of cannabis products to alleviate medical conditions that themselves may cause impairment (MacCallum et al. 2022). This adds to evidence that regular cannabis users are less susceptible to THC-induced impairment than occasional users with an equivalent dose of THC (McCartney et al. 2021b; Bosker et al. 2012; Ramaekers et al. 2009). At the same time, MC patients may perceive less risk around DUIC than other users (Wickens, et al. 2019) leading to a higher likelihood of DUIC (Wickens et al. 2022). It remains to be seen whether the relatively low perception of risk associated with DUIC seen here is an accurate assessment of risk.

Our findings indicate that a significant minority (39%) of people using MC reported driving within 3 h of consuming cannabis products—the time period most likely to be associated with intoxication and driving impairment based on studies of NMC users with limited tolerance to cannabis. The extent to which impairment occurs in people who use MC regularly and develop tolerance to the effects of cannabis remains unclear. Many classes of medications (e.g., opioids, benzodiazepines, antipsychotics, antihistamines, antidepressants) are known to cause impairment, and indeed studies highlight many such medications impair driving to a similar or greater extent than cannabis (Arkell et al. 2021). Most countries have driving policies recognising that such medications can cause drowsiness and impairment, and so patients are cautioned against driving if they are experiencing impairment. Clinically, health care professionals caution patients about driving if impaired, particularly so when commencing treatment and stabilizing on their dose, or when there are significant dose increases. It has been argued that a similar approach should be applied to patients prescribed MC in place of the blanket prohibition in Australia which prevents MC users from driving altogether. Further research examining driving impairment in patients prescribed MC long term would provide critical evidence in this debate and is sorely needed.

While evidence for sex differences in acute cannabis effects is conflicting [e.g., (Sholler et al. 2021; Arkell et al. 2022; Matheson et al. 2020)], an analysis of data from the 2016–2017 National Survey on Drug Use and Health in the US indicated a significantly greater probability of DUIC among males than females for both combined MC/NMC users and NMC-only users (Lloyd et al. 2020). Overall, the probability of DUIC ranged from 20 to 25% for females and from 28 to 40% for males, suggesting a possible need for more targeted interventions (Lloyd et al. 2020). A recent study by Wickens et al. (Wickens et al. 2022) likewise observed a greater incidence of DUIC among males relative to females (20.7 vs. 8%). No such difference was observed in the present analysis, although it is important to note that our survey only included MC users while these other two studies included both MC and NMC users. Further work might seek to elucidate whether sex differences do exist in DUIC likelihood among broader groups of patients.

This study is not without limitations. Self-report data are inevitably prone to inaccuracies and response bias. The use of convenience sampling may have also introduced a selection bias toward respondents who favor the relaxation of stringent cannabis and driving laws. Nonetheless, we were able to capture a wide range of responses from individuals using a variety of products, cannabinoid formulations, and routes of administration. As noted elsewhere (Lintzeris et al. 2022), demographic data in the prescribed group here are similar to the broader demographic data from the Australian regulator around patients receiving MC prescriptions, suggesting that this cohort is a good representation of the community at large.

## Conclusions

The present study captures attitudes and perceptions toward DUIC among a diverse group of Australian MC users. Of those who reported driving a motor vehicle in the past 12 months, 28% reported DUIC, and 49–55% (depending on the route of administration) reported driving within 6 h of cannabis use. Binary logistic regression highlighted several factors that may increase the likelihood of DUIC and should be considered when developing consumer education, clinical guidance, and road safety policy. A key priority for future research is to investigate the effects of prescribed MC on driving ability and cognitive function in patient populations.

### Supplementary Information

**Additional file 1: Table S1.** Questionnaire.

## Data Availability

The survey questions are available as an online supplement. The data sets used and/or analysed during the current study are available from the corresponding author on request.
