# Supplementary material for: Driving-related behaviors, attitudes, and perceptions among Australian medical cannabis users: results from the CAMS 20 survey
Source: J Cannabis Res. 2023 Sep 6;5:35. doi: 10.1186/s42238-023-00202-y (PMC10481606; doi:10.1186/s42238-023-00202-y)
Supplement: Supplementary file 1 — Additional file 1: Table S1. Questionnaire. [file 42238_2023_202_MOESM1_ESM.pdf]

SECTION 9

DRIVING

Have you driven a motor vehicle in the past 12 months?	◯ Yes<br>◯ No
Have you EVER been convicted of driving under the influence of cannabis?	◯ Yes<br>◯ No
After using medicinal cannabis, how long do you typically wait before driving?	◯ I drive within one hour of using cannabis<br>◯ I wait 1-3 hours after using cannabis<br>◯ I wait 4-6 hours after using cannabis before driving<br>◯ I wait 7-12 hours after using cannabis before driving<br>◯ I wait 13-24 hours after using cannabis before driving<br>◯ I wait at least 24 hours after using cannabis before driving
In the past 12 months, did you ever drive while under the influence of cannabis (i.e. while you were high)?	◯ Yes<br>◯ No
In the past 12 months, how often did you drive while under the influence of cannabis (i.e. while you were high)?	◯ Very rarely<br>◯ Rarely<br>◯ Sometimes<br>◯ Often<br>◯ Very often
In the last 12 months, have you undergone road side testing by the police when having used medicinal cannabis?	◯ Yes<br>◯ No
Does the presence of roadside drug testing deter you from driving after you have consumed medicinal cannabis?	◯ Yes<br>◯ No
Have you been convicted of driving under the influence of cannabis IN THE LAST 12 MONTHS?	◯ Yes<br>◯ No
Q 4.19 skipped	
Have you EVER been convicted of driving under the influence of cannabis?	◯ Yes<br>◯ No

Thinking about how you feel after consuming medicinal cannabis, please indicate to what extent you agree or disagree with the following statements:

I am slower to react to sudden situations	◯ Strongly Disagree<br>◯ Disagree<br>◯ Neither agree nor disagree<br>◯ Agree<br>◯ Strongly agree

I find it harder to remain focused	◯ Strongly Disagree<br>◯ Disagree<br>◯ Neither agree nor disagree<br>◯ Agree<br>◯ Strongly agree
I tend to drive more carefully	◯ Strongly Disagree<br>◯ Disagree<br>◯ Neither agree nor disagree<br>◯ Agree<br>◯ Strongly agree
I find it harder to stick to the speed limit	◯ Strongly Disagree<br>◯ Disagree<br>◯ Neither agree nor disagree<br>◯ Agree<br>◯ Strongly agree
I tend to leave a larger gap between me and the car ahead	◯ Strongly Disagree<br>◯ Disagree<br>◯ Neither agree nor disagree<br>◯ Agree<br>◯ Strongly agree
I find myself taking more risks	◯ Strongly Disagree<br>◯ Disagree<br>◯ Neither agree nor disagree<br>◯ Agree<br>◯ Strongly agree
I find it harder to drive in a straight line	◯ Strongly Disagree<br>◯ Disagree<br>◯ Neither agree nor disagree<br>◯ Agree<br>◯ Strongly agree
I feel more in control of the vehicle	◯ Strongly Disagree<br>◯ Disagree<br>◯ Neither agree nor disagree<br>◯ Agree<br>◯ Strongly agree
I can accurately assess my driving ability	◯ Strongly Disagree<br>◯ Disagree<br>◯ Neither agree nor disagree<br>◯ Agree<br>◯ Strongly agree
Do you think medicinal cannabis impairs your driving ability?	◯ Yes<br>◯ No<br>(select one option)
Do you think non-medical ('recreational') cannabis impairs your driving ability?	◯ Yes<br>◯ No
